# Quorum sensing systems and related virulence factors in *Pseudomonas aeruginosa* isolated from chicken meat and ground beef

**DOI:** 10.1038/s41598-021-94906-x

**Published:** 2021-08-02

**Authors:** Gökhan İnat, Belgin Sırıken, Ceren Başkan, İrfan Erol, Tuba Yıldırım, Alper Çiftci

**Affiliations:** 1 Department of Food Hygiene and Technology, Faculty of Veterinary Medicine, Ondokuz Mayıs University, Samsun, Turkey; 2 Department of Aquatic Animal Diseases, Faculty of Veterinary Medicine, Ondokuz Mayıs University, Samsun, Turkey; 3 Department of Physical Therapy and Rehabilitation, Sabuncuoğlu Şerefeddin Health Services Vocational School, Amasya University, Amasya, Turkey; 4 Faculty of Health Sciences, Eastern Mediterranean University, Gazimagusa TRNC Via Mersin, Turkey; 5 Department of Biology, Faculty of Arts and Sciences, Amasya University, Amasya, Turkey; 6 Department of Microbiology, Faculty of Veterinary Medicine, Ondokuz Mayıs University, Samsun, Turkey

**Keywords:** Molecular biology, Microbiology, Bacteria, Biofilms, Microbial genetics, Pathogens

## Abstract

The objective of this study was to evaluate 50 [chicken meat (n = 45) and ground beef (n = 5)] *Pseudomonas aeruginosa* isolates to determine the expression of the lasI and rhl QS systems, related virulence factors, and the presence of *N*-3-oxo-dodecanoyl homoserine lactone (AHL: 3-O-C_12_-HSL). For the isolation and identification of *P. aeruginosa*, conventional culture and *oprL* gene-based molecular techniques were used. In relation to QS systems, eight genes consisting of four intact and four internal (*lasI/R*, *rhlI/R*) genes were analyzed with PCR assay. The two QS systems genes in *P. aeruginosa* isolates from ground beef (80.00%) and chicken meat (76.00%) were present at quite high levels. The 3-O-C_12_-HSL was detected in 14.00% of the isolates. Both biofilm formation and motility were detected in 98.00% of the isolates. Protease activity was determined in 54.00% of the isolates. Pyocyanin production was detected in 48.00% of the isolates. The las system scores strongly and positively correlated with the rhl system (*p* ˂ .01). PYA moderately and positively correlated with protease (*p* ˂ .05). In addition, there was statistically significance between *lasI* and protease activity (*p* < .10), and *rhlI* and twitching motility (*p* < .10). In conclusion, the high number of isolates having QS systems and related virulence factors are critical for public health. Pyocyanin, protease, and biofilm formation can cause spoilage and play essential role in food spoilage and food safety.

## Introduction

*P. aeruginosa* is an opportunistic Gram-negative pathogenic bacterium for human and animals^1^. It is also a major food spoilage bacterium due to its proteolytic and lipolytic activities, off-flavors, and pigment secretion features^2^. Factors of bacterium-related pathogenicity and food spoilage are generally regulated by the QS system^3^.

The QS system is a communication process among bacteria when they are exposed to unfavourable environmental conditions^4^. For communication, bacteria use low density, small, and diffusible signal molecules called autoinducers (AIs)^5^. There are four types of QS systems in *P. aeruginosa,* including las, rhl and *Pseudomonas* quinolone signal (PQS)^6^, and these systems provide bacteria to govern their species-specific and/or inter-species synchronized group behaviours^7,8^.

Las and rhl QS systems are composed of three essential factors: i) signal synthase (*I* gene), which is responsible for synthases of AI signal molecules; ii) signal receptor (*R* gene), which is necessary for coding the transcriptional activator protein (R protein); and iii) signal molecules (AIs). In *P. aeruginosa*, AIs are generally *N*-acyl-homoserine lactone (AHL) molecules^6^, and the *P. aeruginosa* genome encodes mainly two types of AHL: 3-O-C_12_-HSL for the las QS system and *N*-butanoyl-homoserine lactone (C_4_-HSL) for the rhl QS system^9^.

Expression of virulence genes, biofilm formation and motility play crucial roles in the pathogenesis of *P. aeruginosa.* These factors regulated by QS systems are also critical for withstanding stressful conditions^6,8,10^. Protease activity and pyocyanin (PYA) production are two of these virulence factors. PYA plays an important role in pathogenesis of *P. aeruginosa*, such as stimulating apoptosis in neutrophils, suppressing the host response, and increasing IL-8^11^. PYA synthesis process begins with the accumulation of necessary signal molecules on a cell-dependent fashion^7^. Protease is another important pathogenic factor, and 3% of the whole genome of *P. aeruginosa* encodes protease^12^. *P. aeruginosa* has three types of motility, including swimming, swarming, and twitching^13^. Biofilm formation also plays a significant role in *P. aeruginosa* infections^14^. The biofilm formation consists in many phases, and QS systems appear to be involved in all biofilm formation steps^15^, such as microbial surface attachment, exopolysaccharide matrix production, and biofilm detachment or degradation^16^. Motility and PYA production are also related to different steps of biofilm formation^17,18^.

*P. aeruginosa* is an important nosocomial infectious agent^1^, and the QS system plays a significant role in its pathogenesis because it controls and designs the expression of many virulence factors^7,8^. The extracellular enzymatic activities of the bacterium regulated by the QS system also plays a major function in food spoilage^19^. The QS system and the related virulence factors of *P. aeruginosa* have been extensively studied in human origin isolates^6,14^; however, there seems to be inadequate scientific data on food isolates. Therefore, the objective of this study is to evaluate 50 *P. aeruginosa* isolates obtained from chicken meat and ground beef sold in the Samsun province of Turkey to determine the expression of the two QS systems, AHL (3-O-C_12_-HSL), and certain QS system-related virulence factors, including biofilm formation, motility, protease activity, and PYA production.

## Results

The results of the study are depicted in Table 2. According to the findings, 42 (84.00%) out of 50 isolates had at least one of the two QS systems’ genes, and all four genes (*lasI/R* and *rh1I/R*) belonging to the two QS systems were detected in 10 (20.00%) isolates. In addition, both one of las (*lasI* or/and *lasR*) and rhl (*rhlI* or/and *rhlR* genes) systems genes were detected in 35 (70.00%) of the isolates (four ground beef and 31 chicken beef sample).

Based on las and rhl QS systems genes, *lasI* and *lasR* genes were detected in 36 (72.00%) and 28 (56.00%) of the isolates, respectively. Furthermore, *rhlI* and *rhlR* genes were detected in 23 (46.00%) and 34 (68.00%) of the isolates, respectively.

There were differences in terms of the presence of intact and internal genes: namely, the number of internal genes (n = 106) outnumbered intact genes (n = 43). In terms of gene levels, intact *lasI/R* and *rhl/R* genes were determined in 19 (12/7) and 24 (14/10) of the isolates, respectively. Internal *lasI/R* and *rhlI/R* genes were determined in 58 (33/25) and 48 (16/32) of the isolates, respectively (Table 2). In five isolates, only intact *lasI/R* (2/3) genes were detected, and in nine isolates, only intact *rhlI/R* (7/2) genes were detected.

In the study, 3-O-C_12_-HSL was detected in seven (14.00%) of the isolates belonging to only chicken meat. However, las system genes were not detected in the two of seven isolates. For this reason, it was used the new primer set as seen in method section and Table 1. It was detected only *lasI* gene in the all AHL (3-O-C_12_-HSL) positive isolates in contrast to *lasR* gene.

**Table 1:** Primers used in this study.

Amplified	Oligonucleotide sequence 5′ → 3′	Product size (bp)	References
**Species-specific**			
*oprL F*	ATGGAAATGCTGAAATTCGGC	504	De Vos et al.^21^
*oprL R*	CTTCTTCAGCTCGA CGCGACG		
**QS intact genes**			
*lasI* F	ATGATCGTACAAATTGGTCGGC	605	Schaber et al.^23^
*lasI* R	GTCATGAAACCGCCAGTCG		
*lasR* F	ATGGCCTTGGTTGACGGTT	725	
*lasR* R	GCAAGATCAGAGAGTAATAAGACCCA		
*rhlI* F	CTTGGTCATGATCGAATTGCTC	625	
*rhlI* R	ACGGCTGACGACCTCACAC		
*rhlR* F	CAATGAGGAATGACGGAGGC	730	
*rhlR* R	CTTCAGATGAGGCCCAGC		
**QS internal genes**			
*lasI* F	TCGACGAGATGGAAATCGATG	363	Schaber et al.^23^
*lasI* R	GCTCGATGCCGATCTTCAG		
*lasR* F	TGCCGATTTTCTGGGAACC	362	
*lasR* R	CCGCCGAATATTTCCCATATG		
*rhlI* F	CGAATTGCTCTCTGAATCGCT	143	
*rhlI* R	GGCTCATGGCGACGATGTA		
*rhlR* F	TCGATTACTACGCCTATGGCG	207	
*rhlR* R	TTCCAGAGCATCCGGCTCT		
*lasI* F	CGTGCTCAAGTGTTCAAGG	295	Zhu et al.^46^^a^
*lasI* R	TACAGTCGGAAAAGCCCAG		
*lasR* F	AAGTGGAAAATTGGAGTGGAG	139	
*lasR* R	RGTAGTTGCCGACGACGATGAAG		

^a^It was applied only for AHL positive isolates.

Biofilm formation was detected in 49 (98.00%) of the isolates using one of the three methods: namely, 40 (80.00%) by CRA method, 28 (56.00%) by used microtiter plate, and 42 (84.00%) by the test tube method. In addition, 17 (34.00%) and 22 (44.00%) of the isolates demonstrated biofilm formation capability when using two and three different biofilm formation detecting methods in the study. When comparing the three methods in terms of biofilm formation, the test tube method was superior to the other two methods. Regarding to sample types, four out of five ground beef isolates were capable of biofilm formation at least two or three biofilm detection methods. For chicken meat samples, 16 (32.00%) and 19 (38.00%) isolates were determined in biofilm formation when using two and three methods, respectively (Table 2). According to the OD values of the isolates obtained from microtiter plate test results, the OD values of 28 biofilm-positive isolates ranged from 0. 3250 to 1.2035, and 5 of 28 isolates demonstrated higher OD values (> 0.4580) than the reference strain.

**Table 2:** HSL, the QS-system genes, biofilm formation, motility, PYA, protease and elastase activity test results of the 50 *P. aeruginosa* isolates.

Isolate no	3-O-C_12_ HSL	The QS systems								Biofilm formation (n = 49, 98.00%)			Motility (n = 49, 98.00%)			Virulence factors	
		las (n = 38, 76.00%)				rh1 (n = 37, 74.00%)				CRA	Mic. plate	Tube	Twitching	Swarming	Swimming	PYA	Protease
		Intact gene (bp)				Internal gene (bp)											
		*lasI 605*	*lasR* 725	*rh1I* 625	*rh1R* 730	*lasI 363*	*lasR 362*	*rh1I* 143	*rh1R* 202								
1^Cm^	−	−	−	−	−	−	−	−	−	+	+	+	−	−	+	+	−
2 ^Cm^	−	−	−	−	−	+	−	−	−	+	+	+	−	−	+	−	+
3 ^Gb^	−	−	−	−	−	+	+	−	+	+	+	+	+	−	+	+	−
4 ^Cm^	−	−	−	−	−	+	+	−	+	+	−	+	+	−	−	+	+
5 ^Cm^	−	−	−	+	−	+	+	−	+	+	+	+	−	−	+	+	+
6 ^Cm^	−	−	−	−	+	−	+	−	+	+	+	−	+	−	+	−	−
7 ^Cm^	+	−	−	−	−	+*	−	+	+	+	−	+	−	−	+	−	−
8 ^Cm^	+	−	−	−	−	+*	−	−	−	+	−	+	−	−	+	+	+
9 ^Cm^	−	−	−	−	−	−	−	−	−	−	−	+	−	−	+	+	+
10 ^Cm^	+	−	−	−	+	+	+	+	+	+	+	−	−	−	+	+	−
11 ^Cm^	+	−	−	−	−	+	+	+	+	+	−	−	−	−	+	+	−
12 ^Cm^	+	+	−	+	+	−	−	+	+	+	−	−	−	−	+	+	+
13 ^Cm^	−	−	−	−	−	−	−	−	−	+	−	+	−	−	+	−	−
14 ^Cm^	−	−	+	−	−	−	+	+	+	−	−	+	−	+	+	−	−
15 ^Cm^	−	−	−	−	−	+	−	−	−	+	−	−	+	−	+	−	+
16 ^Cm^	−	−	−	−	−	+	+	−	+	+	+	−	+	−	+	−	+
17 ^Cm^	+	−	+	+	−	−	−	−	+	+	+	+	−	−	+	−	−
18 ^Gb^	−	−	−	−	−	+	+	−	+	+	+	+	+	−	+	+	+
19 ^Cm^	−	−	−	+	−	+	+	+	+	+	+	+	−	−	−	−	−
20 ^Cm^	−	−	−	−	−	+	+	+	+	−	−	−	−	−	+	+	+
21 ^Cm^	−	−	−	−	−	−	−	+	−	+	+	+	−	−	+	+	+
22 ^Cm^	−	−	−	−	+	+	+	−	+	+	+	+	+	−	+	+	+
23 ^Cm^	−	−	−	−	−	−	−	+	−	+	−	+	+	−	+	+	+
24 ^Cm^	−	−	−	−	−	−	−	−	−	+	+	+	−	−	+	−	−
25 ^Cm^	−	−	−	−	−	−	−	−	−	+	+	+	+	−	+	−	+
26 ^Cm^	−	+	+	+	−	+	+	−	+	+	−	+	−	−	+	−	+
27 ^Cm^	−	−	−	−	−	−	−	−	−	+	+	+	−	−	+	−	−
28 ^Cm^	−	+	−	+	+	+	−	−	+	+	+	+	+	−	+	+	+
29 ^Cm^	−	−	−	−	−	−	+	−	−	+	+	+	+	−	+	+	−
30 ^Cm^	−	−	−	−	−	+	+	−	+	+	−	+	−	−	+	+	+
31 ^Cm^	−	−	−	+	+	+	−	+	+	−	−	+	−	−	+	+	+
32 ^Cm^	−	+	−	−	−	+	+	−	+	−	−	+	−	−	+	+	+
33 ^Cm^	−	+	−	+	−	+	−	+	+	+	+	+	+	−	+	−	−
34 ^Cm^	−	−	+	−	+	+	+	−	+	+	+	+	+	−	+	−	−
35 ^Cm^	−	−	−	−	−	−	−	−	−	+	+	−	−	−	+	+	−
36 ^Cm^	−	+	−	+	−	+	+	−	+	+	−	+	+	−	+	−	−
37 ^Cm^	−	−	+	+	+	+	−	+	−	−	−	+	−	−	+	−	−
38 ^Cm^	−	−	+	−	+	+	−	−	−	+	+	+	−	+	+	+	+
39 ^Cm^	−	−	−	−	−	−	−	−	+	−	−	+	+	−	−	−	−
40 ^Cm^	−	−	−	+	−	+	+	−	+	+	+	+	−	−	+	+	+
41 ^Cm^	−	−	−	+	−	+	+	−	−	+	+	+	−	−	+	−	+
42 ^Cm^	+	−	−	−	−	+	+	+	+	−	+	+	−	+	+	+	+
43 ^Cm^	−	+	−	+	−	+	−	+	+	+	−	+	−	−	+	+	−
44 ^Cm^	−	+	−	−	+	+	−	−	+	+	+	+	−	−	+	−	+
45 ^Gb^	−	−	−	−	−	−	−	−	−	+	−	+	+	−	+	−	+
46 ^Cm^	−	+	−	−	−	−	−	−	−	+	+	+	−	−	+	−	−
47 ^Gb^	−	+	−	−	−	+	+	−	+	+	+	+	+	−	+	−	+
48 ^Gb^	−	+	−	−	−	+	+	−	+	−	−	+	+	+	+	−	+
49 ^Cm^	−	−	+	+	−	+	+	+	+	+	−	+	+	−	+	−	−
50 ^Cm^	−	+	−	−	−	+	+	+	+	−	+	+	+	−	+	−	−
	7 (%) (14.00)	12 (%) (24.00)	7 (%) (14.00)	14 (%) (28.00)	10 (%) (20.00)	33 (%) (66.00)	25 (%) (50.00)	16 (%) (32.00)	32 (%) (64.00)	40 (%) (80.00)	28 (%) (56.00)	42 (%) (84.00)	20 (%) (40.00)	4 (%) (8.00)	47 (%) (94.00)	24 (%) (48.00)	27 (%) (54.00)

^Cm^: Chicken meat; ^Gb^: Ground beef; *Based on the according to third primer set described by Zhu et al.^46^.

Motility was detected in 49 (98.00%) isolates using at least one method. In regard to motility types, swimming, twitching, and swarming types of motility were detected in 47 (94.00%), 20 (40.00%), and 4 (8.00%) of the isolates, respectively. Twenty-eight (46.00%) of the isolates demonstrated one type of motility, and 20 (40.00%) of the isolates displayed two types of motility. The remaining one isolate exhibited all three types of motility (Table 2).

With regard to other QS-dependent virulence factors test results, in the present study, it was found that 24 (48.00%) and 27 (54.00%) of the isolates were capable of PYA production and protease activity, respectively (Table 2).

Results of Pearson product-moment correlation analyses suggested that las system scores strongly and positively correlated with rhl system (*r* = 0.52, *p* ˂ 0.01). Similarly, *lasR* strongly and positively related to *rhIR* scores (*r* = 0.56, *p* ˂ 0.01). *rhlI* moderately and positively correlated with *rhlR* (*r* = 0.34, *p* ˂ 0.05). Lastly, PYA moderately and positively correlated with protease (*r* = 0.32, *p* ˂ 0.05). According to Pearson and Fisher’s exact chi square test analyses results, statistically significance was between *lasI* and protease activity (*p* value = 0.07615; 0.05 <  = *p* < 0.10), and *rhlI* and twitching motility (*p* value = 0.0858; 0.05 <  = *p* < 0.10).

## Discussion

In contrast to foods and animal origin isolates, there have been some studies on QS systems and the relationship between QS systems and the related virulence factors of *P. aeruginosa* from human clinical isolates^20^. Therefore, the results of the present study were primarily evaluated in light of data obtained from human clinical *P. aeruginosa* isolates.

In the present study, four las and rhl QS systems genes were detected together in 20.00% of the isolates. In addition, 84.00% of the isolates had at least one of four QS system genes (Table 2). The results clearly indicate that the presence of the two QS systems’ genes in *P. aeruginosa* isolates from ground beef (80.00%) and chicken meat (78.00%) was at quite a high level.

According to the las QS system genes results, the number of *lasI* genes exceeded that of the *lasR* gene. This result was expected as the *lasI* gene first regulates the 3-O-C_12_-HSL signal molecule synthesis. Then, the signal molecules bind to its cognate receptor LasR. Later, the LasR-3-O-C_12_-HSL complex induces to express many target genes^21^. For the activation of LasR, a threshold concentration level of 3-O-C_12_-HSL is required. Thus, *lasI* presence does not always mean that it will be present in *lasR*^22^.

In this study, *rhlI* and *rhlR* genes were detected in 46.00% and 68.00% of the isolates, respectively. LasR-3-O-C_12_-HSL complex regulates *rhlR* gene expression. Therefore, the las system is necessary for the activation of the rhl system^23^. According to statistical analyses results, there is strongly positive correlation between the las system and the rhl system (*p* ˂ 0.01).

In this study, differences between the numbers of intact and internal genes were detected, and the total internal gene number was higher than intact gene numbers, which is in agreement with other study results^24,25^.

In the present study, 3-O-C_12_-HSL as a signal molecule of las QS system was found only in seven (14.00%) isolates. At least one (*lasI*) of las system genes was detected in the seven isolates. The results revealed seven AHL-positive isolates: two of them were capable of biofilm formation, the swimming type of motility, and PYA production. Furthermore, three isolates were capable of biofilm formation, swimming type of motility, PYA production, and protease activity, and two isolates had biofilm formation and swimming type of motility.

Motility is also strongly associated with *P. aeruginosa* pathogenesis^26^*.* In the present study, most of the isolates (98.00%) were capable of at least one of the three motility types. Twitching motility enables a solid surface movement in *P. aeruginosa*. The bacteria can move in a liquid environment using swimming motility mediated by a single polar flagellum^27^. As such, in the present study, 36.00% of the isolates may be capable of both liquid and solid surface movement due to having both twitching and swimming types of motility.

The near surface movement twitching and swimming of *P. aeruginosa* have also been associated with various virulence factors and biofilm formation^28^. The hypothesis is supported by the present study findings. For instance, 46 in 47 isolates possessing swimming ability were also able to form biofilms. In addition, in the present study, a total 26 isolates having swimming motility were also capable of proteolytic activity, and 23 isolates were able to produce pyocyanin.

In the present study, there was a correlation between the two QS systems and motility types. Related to statistical analyses, there was statistically significance between *rh1I* and twitching motility (*p* < 0.10). According to Turkina and Vikstrom^29^, *rhlR* regulates the swarming type of motility. Similarly, in the present study, the *rhlI* gene was detected in all four isolates that had swarming motility. In addition, according to Glessner et al.^30^, the las and rhl QS systems are required for twitching motility in *P. aeruginosa.* In this respect, las, rhl or both of the two QS systems’ genes were detected in 18 out of 20 twitching motility-positive isolates. However, none of the two QS systems’ genes were detected in the one twitching motility isolate. The situation can be explained by according to Beatson et al.’s^31^ study results. The researchers found that when the las or rhl QS systems genes were not activated for gene expression or lacking, *vfr* and *algR* genes also encodes the required proteins for twitching motility.

With respect to QS systems and biofilm formation, most of the isolates exhibited biofilm formation (98.00%), and both las and rhl QS systems genes were present in 72% of the isolates, which indicates that *lasI/R* and *rhlI/R* genes were involved in biofilm formation. Likewise, Davies et al.^32^ reported that the QS signal molecule (3-O-C_12_-HSL) was necessary for normal biofilm structure and differentiation. According to another study O’Toole and Kolter^17^ reported that *lasI* mutation in *P. aeruginosa* resulted in a lack of biofilms. Regarding the past literature, the present study results differed to some extent. Hence, it was demonstrated that the las QS system was absent but rhl QS system was presents in one isolates, which were also capable of biofilm formation. Similarly, it was also reported that *rhlI* gene assisted in biofilm formation and that the Rhl-HSL molecule complex played a crucial role in this aim^33^. In the present study, both the two QS systems’ genes and 3-O-C_12_-HSL product were not determined in nine isolates which had biofilm formation. In these nine isolates with biofilm formation, other factors such as twitching and swimming motility or PYA production might contribute to biofilm formations. Four of the nine isolates were capable of both PYA production and swimming motility. However, two studies demonstrated opposite results regarding correlation between PYA production and biofilm formation. According to the two studies’ results, there was a negative correlation between PYA production and biofilm formation as well as PYA production and swimming type motility^34,35^. In agreement with our findings, Macin^26^ found a positive correlation between pigment production and isolate motility (*p* ≤ 0.05).

The motility types can also change according to biofilm-formation stages. For example, during the initial stage of biofilm formation, the swimming type of motility plays a key role^35^. However, late stage of biofilm architecture, twitching type motility required for dispersing to near surface^36^ and the bacteria require for swarming type of motility^33^. When the present study findings are evaluated in these respects, 47 in 49 biofilm positive isolates can play a role in the initial biofilm formation stage due to their swimming motility. After biofilm formation, 20 of them could be dispersed near the surface due to their twitching motility in the isolates, and one of them formed a harder biofilm architect.

Another virulence factor regulated by QS systems of *P. aeruginosa* is proteolytic activity because the bacteria lead to tissue destruction, invasion ability, and easy adaptation to different environment conditions using protease production^26^.

There have been some studies suggesting a positive correlation between the two QS systems and the proteolytic activity of *P. aeruginosa* isolates^37–39^. The present study results support this view to a significant extent, and 54.00% of the 50 *P. aeruginosa* isolates were found to have protease activity, but only three isolates had neither las nor rhl QS system genes. Similarly, Tingpej et al.^38^ analyzed 43 human origin *P. aeruginosa* isolates for the presence of *lasI* and *lasR* genes as well as for protease activity. They found that *lasI* and *lasR* genes were detected in 42 out of 43 isolates and that 37 (86%) of 43 isolates displayed protease activity. In addition, *rhlI* and *rhlR* genes were also detected in all 43 isolates. Thirty-two isolates had at least one AHL, and *rhl*-associated AHL detection was related to total protease activity and elastase activity (*p* < 0.01). In the present study, 3-O-C_12_-HSL was detected in three protease-activity positive isolates. However, *lasI* and *lasR* genes in combination with each other were determined in 16 isolates, and *rhlI* and *rhlR* genes in combination were found in five isolates. Beside these, only *lasI* and *lasR* were determined in 21 and 17 isolates, respectively. The present study suggests that the las QS system plays an important role (*p* < 0.10), for protease production compared to the rhl QS system.

Some studies have demonstrated that QS also plays a role in spoilage and slime production^19^. Similarly, Liu et al.^19^ demonstrated when *Pseudomonas* concentrations reached about 10^8^ to 10^9^ CFU/g, detectable AHL concentrations appeared on meat surface^40^. In addition, when the bacteria concentration is reached approximately 10^8^ CFU/g, detectable spoilage reactions such as pigment production and slime formation were observed. The QS system-related protease activity for *P. aeruginosa* also plays a significant role in the spoilage processes^41^. During the enzymatic breakdown of amino acids, volatile compounds are released, and the production of volatile compounds is a sign of organoleptic degradation in meat spoilage^42^. Considering this phenomenon, chicken meat and ground beef origin *P. aeruginosa* isolates with PYA production, protease activity, and biofilm formation can cause spoilage and economic loss.

According to the findings of the present study, tested *P. aeruginosa* isolates have some virulence factors regulated by the QS systems, which play an important role in pathogenesis of the bacterium. Therefore, all precautionary measures, such as reducing contamination, controlling growth conditions of the bacterium in poultry and meat processing plants, and using QS system inhibitors, should be taken to avoid biofilm formation and spoilage reaction in chicken meat and ground beef.

## Material and methods

### Sample collection

In the present study, chicken (n = 50) and ground beef (n = 30) samples purchased from supermarkets and butchers in Samsun Province in Turkey in 2018 were analyzed to detect the presence of *P. aeruginosa*.

### Detection of the isolates

The conventional culture technique was used for the isolation of *P. aeruginosa*. For this purpose, Pseudomonas CN Selective Agar [Oxoid SR 102E, suppl. Pseudomonas Agar base-(Oxoid)] for isolation, and Tryptone Soya Agar (TSA-Oxoid-CM0131-L21) for subculturing and identification tests (Gram staining, oxidase, and catalase tests) were used^40^. Molecular confirmation of the isolates that targeted *P. aeruginosa* species-specific *oprL* gene region was performed using the PCR technique^43,44^. A total of 200 *Pseudomonas* spp. isolates were obtained from the samples; among them, 140 isolates were identified as *P. aeruginosa* using the conventional culture technique. However, 50 out of 140 isolates were confirmed by PCR. Only PCR confirmed 50 (45 chicken meat and 5 ground beef origin samples) *P. aeruginosa* isolates obtained from 46 samples (42 of chicken meat and 4 of ground beef samples) were stored at − 80 °C in cryovials containing 10% (w/v) glycerol in a brain heart infusion broth for further analysis.

For determination of QS systems-related genes, AHL signal molecules, virulence factors, and motility, *P. aeruginosa* (ATCC 15692) and *E. coli* (ATCC 25922) strains were used as positive and negative controls, respectively. For biofilm formation, *P. aeruginosa* ATCC 15692 strain was used as a positive control.

### Detection of QS genes using PCR assay

The eight gene regions related to the las and rh1 QS-systems, which consisted of four intact (*lasI/R, rhlI/R*) and four internal (*lasI/R, rhlI/R*) genes, were analyzed in the isolates using a single target PCR assay, according to Schaber et al.^45^. The use of two PCR assays for intact and internal genes is necessary due to considerable variability in the entire operon genes. The oligonucleotide primers and their product sizes used in this study are listed in Table 1.

PCR conditions of four internal genes were as follows: an initial denaturation step at 95 °C for 5 min, followed by 32 cycles of denaturation at 95 °C for 30 s, annealing at 59 °C for 60 s, extension at 72 °C for 90 s, and a final extension step at 72 °C for 10 min.

Amplification conditions of four intact genes included initial denaturation at 95 °C for 5 min, which was followed by 34 cycles at 94 °C for 30 s, at 50 °C for 30 s and 72 °C for 2 min. A final extension step at 72 °C for 10 min was also employed.

In addition, PCR assay was applied according to the method described by Zhu et al.^46^ for *lasI/R* genes detection in AHL positive seven isolates. The method was slightly modified for annealing temperature (57 instead of 52) and MgCl_2_ levels (1.5 mM MgCl_2_ instead of 2.5 mM) to improve the results. The new primer design has shown in Table 1.

### Cross-feeding test for the detection of AHL (3-O-C_12_-HSL)

AHL (3-O-C_12_-HSL) molecule was determined and evaluated according to the method described by Shaw et al.^47^, Ravn et al. ^20^, and Kumar et al.^48^. For the test, 5-bromo-4-chloro-3-indolyl-β-D-galactopyranoside (X-gal, Sigma) was prepared (20 mg of X-gal in 1 mL of dimethyl sulfoxide [DMSO]). Then, 40 μL of X-gal-DMSO solution was spread onto a Luria–Bertani (LB) agar plate and incubated at 37 °C for 1–2 h for drying. Next, an *Agrobacterium tumefaciens* ATCC-51350, strain A136 (updated scientific name as *Rhizobium radiobacter*) reference strain, was inoculated as a line. Then, the isolates were inoculated at 1 cm intervals. The plates were incubated at 37 °C for 48–72 h. Isolates showing greenish-blue pigmentation on the LB agar were evaluated as positive.

### QS-related virulence factors

#### Pyocyanin production

The test was applied according to the method previously described by Schaber et al.^45^ and Carlsson et al.^49^. For this purpose, pyocyanin broth medium, chloroform, and hydrochloric acid were used.

Preparation of the inoculum. The isolates were grown overnight on nutrient agar (Oxoid, England). Then, the optic density of the inoculum was adjusted to a 0.5 McFarland (∼10^8^ CFU/mL) turbidity standard in physiological saline (0.1%).

For this purpose, 100 µL of the isolate solution prepared as described above was inoculated into the PYA broth medium and incubated at 37 °C for 48 h. Following incubation, it was centrifuged for 20 min at 3000×*g* at 4 °C, and supernatant was collected. A 5 mL of the culture supernatant was transferred to 3 mL of chloroform and mixed. After centrifugation for 3 min at 1200×*g* at room temperature, the chloroform layer (2.5 mL) was transferred to an empty tube. Then, 0.2 mL of HCl (Hydrochloric acid) was added into the tube, and the solution was mixed and centrifuged at 845×*g* for 10 min (Nüve, NF 800, Turkey). Following this, the 0.2 mL of HCl on the top layer was removed. The remaining layer within the tube was used for determination of PYA by measuring OD520 nm (TECAN Infinite F50).

#### Protease activity test

The inoculum preparation was described PYA production section. The test was assayed by inoculating 2 µL of each isolate on a LB agar plate supplemented with 2% sterile skim milk. The plate was incubated at 37 °C for 16–18 h. A clear zone surrounding a bacterial colony on the plate was considered as positive proteolytic activity^50^.

#### Biofilm formation test

Biofilm formation was tested by using three different methods; including the tube method, microtiter plate assay, and the Congo red agar (CRA) test.

Preparation of the inoculum: The isolates were grown overnight on nutrient agar (Oxoid CM0003) at 30 °C. Then, the optical density of the inoculum was adjusted to a 0.5 McFarland (∼10^8^ CFU/mL) turbidity standard in physiological saline solution (0.1%). The bacterial suspension was used for the tube method and for the microtiter plate assay.

Tube method (slime production): Modified Tryptic Soy broth (TSB + 0.25% glucose) was used as described previously by Christensen et al.^51^.

Microtiter plate assay: Quantitative biofilm formation test was conducted using the crystal violet (CV) method in 96-well polystyrene microtiter plates according to Stepanovic et al*.*^52^ and Silvia et al.^53^. Evaluation of biofilm formation was performed according to optical density (OD) values.

Congo red agar (CRA) test: A CRA test was applied according to Freeman et al.^54^, and colonies grown on CRA plates that were dark red or blackish in color were evaluated as biofilm producers.

#### Motility tests

Three different methods, including twitching, swarming, and swimming types of motility, were used for this purpose.

Twitching motility was applied according to Déziel et al.^55^. For medium, 3-mm thick 1% LB agar in the polystyrene petri dishes and 1% CV solution were used.

Swarming motility was applied according to Boles et al.^56^. Semi-solid agar (0.5% agar, 8 g/L nutrient broth, and 5 g/L glucose) was used as a medium.

Swimming motility was performed according to Déziel et al.^55^, and medium containing 1% tryptone, 0.5% NaCl, and 0.3% agar was used.

Twitching, swarming, and swimming motility test results for the isolates were evaluated according to Murray et al.^57^. Any isolate that had a detectable twitching or swimming zone upon visible inspection was regarded as positive, and a swarming zone of > 10% in the *P. aeruginosa* (ATCC 15692) strain was considered positive in the swarming motility assay.

### Statistical analysis

Data were processed and analyzed with SPSS 21 for Windows. Pearson product-moment correlation was used to examine strength and direction of linear relationship between study variables. A significance level of *p* ˂ 0.05 was used in all statistical analyses. Pearson and Fisher’s exact chi square test was also applied.
